# Supplementary material for: Learning curve and surgical outcome of robotic assisted colorectal surgery with ERAS program
Source: Sci Rep. 2022 Nov 29;12:20566. doi: 10.1038/s41598-022-24665-w (PMC9709162; doi:10.1038/s41598-022-24665-w)
Supplement: Supplementary file 1 — Supplementary Information 1. [file 41598_2022_24665_MOESM1_ESM.zip › LSC Raw data-Ñ[▒K/098/H_ñw╝╨░O▒Kñσ.pdf]

正式摘要(電子簽章：000000204203544)

RCC / RCC ☐ 英文標題(注意:無英文姓名-請補病人基本檔!) ☐【讀取】憑證

臺中榮民總醫院

出院病歷摘要

轉入號： 轉出號：

[redacted]

(8)流水編號	
(9)入院日期	109/10/19 (2020) 直腸外科 RCC - 06
(10)轉科日期	109/11/12 (2020) - 呼吸治療 - RCC-06
(11)出院日期	109/11/27 (2020) 住院天數計 39 日

診斷

- 入院 :1. Malignant tumor of rectum, pending surgical intervention
  2. Type 2 DM,poor controlling,HbA1c:11.8 on 106/03, under Glucophage + Novomix30 16U/8U BID using,
  3. Hypertension more than 10 years
  4. Traumatic brain injury s/p cranectomy without sequelae for than 20 years ago
- 出院 :1. Aspiration pneumonia,bilateral with respiratory failure and acute respiratory distress syndrome (ARDS) s/p endotracheal intubation and mechenical ventilation since 2020/10/25 and superimposed with pulmonary edema under ventialtro support s/p Tazocin (2020/10/24~2020/11/09) and Cefipime (2020/11/25~), sputum culture yield E.Coli and Klebsiella Pneumoniae
  2. Malignant tumor of rectum s/p laparoscopic low anterior resection (LAR) with end-to-end anastomosis and segmental resection of ileum(5cm)(20cm proximal to IC valve) with double barrel ileostomy over right lower quadrant (RLQ) on 2020/10/21, pT2N0Mx with stomy in place and stomy oozing on 2020/11/07 s/p ligation and bosmin packing with improvement
  3. Refractory pulmonary edema,suspected coronary artery disease (CAD) with lateral wall T inversion
  4. Adrenal insufficiency under cortisone suppliment since 2020/11/25 (Cortisone level : 12.9 ug/dl on 2020/11/13)
  5. Type 2 Diabetes Mellitus (HbA1C:7.1 on 2020/11/12)

主訴

- Stool with blood noted for about 1 month

病史

- The 76-year-old female had the underlying with
  1.scrub typhus infection c/w acute liver enzyme increasing and rhbdomyolysis s/p Doxycycline from 3/3~3/8
  2.type 2 DM,poor controlling,HbA1c:11.8 on 106/03, under Glucophage + Novomix30 16U/8U BID using,
  3.hypertension more than 10 years
  4.traumatic brain injury s/p cranectomy without sequelae for than 20 years ago
  Bloody stool was noted for about 1 month. SHe had went to 金門H where colonoscopy was done and shwoed adnenocarcinoma 7cm FAV. Then, she was referred to our hospital for further treatment. At our CRS OPD, DRE showed a palpable tumor 5cm FAV. This time,she was admitted for surgical treatment.

體檢發現

-
  - General appearance :
    - easy looking , conscious clear , without cardiopulmonary distress
    - Vital signs自動帶入date 體溫(BT) 脈搏(P) 呼吸(R) 血壓(BP) 身高(BH) 體重(BW) BMI
      20201019 36.1C 106/min 18/min 109/94mmHg 145.4cm 43.6kg 20.6
    - Vital signs: B.P. 109/94 mmHg , P.R. 106 /min , R.R. 18 /min , B.T. 36.1 ℃
  - HEENT :
    - Eyes: no anemic conjunctiva, no icteric sclera, normal EOM
    - ENT: no otorrhea, no rhinorrhea, no erythematous mucosa, no injected tonsil, no deformity
  - Neck: supple, freely movable, trachea midline,no deformity, no engorged jugular vein , no palpable thyroid or lymph node enlargement
  - Chest:
    - Inspection: Grossly normal, symmetric and fully expansion, no accessory muscle used, no operation scar, no spider angiomata, no gynecomastia
    - Palpation: no palpable mass or nodule, no subcutaneous emphysema, no tenderness
    - Ausculation: clear breathing sound
  - Heart:
    - Palpation: no thrill, no heave
    - Percussion: normal heart size
    - Ausculation: regular heart beat, normal S1, S2, no gallop, no murmur
  - Extremities: No deformity, full ranged of motion without limitation, no clubbing finger or nail cyanosis, no pitting edema, no ulcer or pigmentation, no obvious skin lesion, no varicose vein, no bone, joint or muscle tenderness

手術日期及方法

- 手術日期及方法20201021 1. Laparoscopic low anterior resection, end-to-end anastomosis, endo GIA 60-3.5mm*2 and EEA 29mm.
  Bilateral doughnut: intact, Air leak test: done, no air leak.
  2. Segmental resection of ileum(5cm)(20cm proximal to IC valve), with double barrel ileostomy at RLQ.
  3. High ligation (+) above the left colic artery.
  4. Cut margin: proximal 14cm, distal 1.5cm.
  5. Take down of splenic flexure: nil.

住院治療經過

- She was admitted for surgical intervention of resection of rectal cancer on 2020/10/21. After operation, she was sent to ordinary ward for care. Abdominal fullness was ntoed since 2020/10/23 and vomiting occurred. Then desaturation(SPO2 was around 70%) without conscious loss and SPO2 could recover to 90% after ambu bagging and sputum suction. N-G tube insertion was done and much bile like drainage came out via N-G tube. Fever flared up and CXR disclosed RLL pneumonia patch. Antibiotics was shifted to Tazocin and lab data revealed leukopenia, bandemia with elevated lactate and CRP level. Aspiration pneumonia,bilateral with respiratory failure was impressed. Endotracheal tube insertion and ICU admission for advanced care were informed to her family. The refused it and family signed DNR. At 3:00 AM on 2020/10/25, desaturation and below 60% was noted and her family refused ETT intubation. So BiPAP was connected and the SpO2 could barely maintained around 58~60% and around 86% at best. After explantation to her again around 12:00 noon, they withdrawal DNR and intubation with mechanical ventialtion was performed. Then, she was sent to SICU for intensive care on 2020/10/25. Bedside abdominal sonography revealed no ascites or GB distension. During stay at SICU, we kept tazocin for broad spectrum coverage. 12 lead EKG revealed T wave inversion at lead II, III, V2~V5 on 2020/10/27 but CK/CK-MB were within normal range. We consulted cardiologist and ACS was less likely under cardiologist suggestion. She tried feeding since 2020/10/28 without vomiting or other discomforts. We kept chest care and clinical condition improved. However, fever episode was noted on 2020/11/02 night. We kept tazocin for 14 days course. We held aspirin due to occasional oozing of colostomy with anaemia and lower level Hgb(7.3g/dl). Due to still ventilator dependent and difficulty weaning, she was transferred to our RCC on 2020/11/12. During stay at our RCC,we kept sitting up training and avoided fluid overload. Refractory pulmonary edema plus sinus tachycardia and lateral wall diffuse T inversion were still found. We consulted cardiologist again which elective CAG can be proceeded but high risk of bleeding after DAPT use and possible adverse event if DAPT hold during acute phase of post stenting status. Due to still ventilator support, we arranged palliative family meeting on 2020/11/18, family still hesitated tracheastomy and denied CAG intervention. They preferred palliative care instead of invasive procedure. We re-confirmed DNR . New episode of shock along with low grade fever took place on 2020/11/24 afternoon. Suspected relative adrenal insufficiency and superimposed with occult infection. Later, desaturation with exaggerated bilateral lung infiltration was noted. We suspected newly developed pneumonia and superimposed with pulmonary edema. We escalated antibiotics to Cefipime and titration of ventilator setting (adjusted PEEP to 10cmH2O and titrated FiO2 around 50%->40%). Critical status was informed, but family denied hemodialysis nor invasive intervention. We provided inotropic agents and combined with diuretics. The family requested to transfer back to Kingmen Hospital. We provided summary note and copied film. She was transferred back to Kingmen Hospital via airplane on 2020/11/27 by against advise discharge (AAD).

合併症

- ARDS

一般檢查

DATE	TIME	ALB	TP	BIL.T	BIL.D	ALKP	AST	ALT	LDH	CHOL	TG	HDL-C	LDL	UA	NH3	AMY
1091019	1713		8.2										75			
1091020	0915									114	150					

1091021	2009	3.4		0.36	0.16		30	12								
1091024	1922			0.34				8								
1091025	0408	2.7		0.58	0.29	45	29	9								
1091025	1246	2.0		0.41	0.24	31	23	9								
1091025	2003			0.66	0.37											
1091026	0531			0.6	0.3	29	29	8								
1091027	1045									91	336					
1091028	0547			0.91	0.44	57	18	8								
1091030	0509			1.41	0.84		36	7								
1091031	0645			2.03	1.18			8								
1091031	1201	2.9														
1091101	0655			1.69	0.98	292	45	16								
1091102	0509	2.8														
1091104	0630			0.99	0.56		35	17	274							
1091105	0516					426		17								
1091107	0552			1.1	0.5		34	16	232							
1091110	0550	3.5		0.63	0.34		29	23	201							
1091112	0607			0.54	0.30	474	38	25								
1091112	1315	4.0				499	52	30	294							
1091113	0547									157	133					
1091116	0557														53	
1091116	0602			0.53		446		26	196							92
1091117	1227	4.0														
1091119	0718			0.52		429		26	235							
1091123	0630			0.44		367		22	186				75			
1091124	1015								194							
1091125	2028	3.2		0.32		458		63	285							

DATE	TIME	WBC	RBC	HGB	HCT	MCV	PLT	BLST	NEUT	BAND	SEG	LYM	MON	EOS	BAS
1091019	1712	7630	3.00	7.5			363		60.7			32.1	5.8	0.9	0.5
1091021	2009	12310		9.6			290		89.0			7.1	3.4	0.3	0.2
1091023	0858	6490		10.2			282			0.9	79.6	12.6	3.9	3.0	0.0
1091024	1918	1270		10.2			272			16.0	70.0	10.0	4.0	0.0	0.0
1091025	0408	7830	3.94	10.5	33.3	84.5	428		80.1			16.3	2.9	0.4	0.3
1091025	1246	7920	3.29	8.7	28.7	87.2	243			14.0	72.0	5.0	3.0	0.0	0.0
1091025	2000	10810	4.31	11.6	37.4	86.8	301		92.1			5.0	2.2	0.0	0.7
1091026	0531	13610	4.10	11.1	35.6	86.8	255		91.0			5.4	3.1	0.0	0.5
1091027	0618	18820		10.3	31.9		223				90.0	6.0	4.0	0.0	0.0
1091028	0544	15650	3.60	9.6	29.5	81.9	196		89.7			7.9	1.5	0.6	0.3
1091030	0507	14050		8.0	24.6		198			1.0	90.0	5.0	2.0	1.0	0.0
1091031	0645	13550	3.63	9.5			239				81.0	11.0	4.0	2.0	0.0
1091101	0656	14760	3.66	9.4	28.6	78.1	338		85.3			8.3	5.1	1.1	0.2
1091102	0509	12580		8.4	25.8		384		82.2			9.1	7.4	1.1	0.2
1091104	0615	13350		8.3	25.8		598		82.7			8.5	7.2	1.2	0.4
1091105	0511	12020		8.4	26.4		652		79.0			12.7	6.5	1.2	0.6
1091106	0511			8.9	27.8		722								
1091107	0551	13160		7.3	23.0		591		77.6			15.6	4.6	1.4	0.8
1091108	0507			9.7	29.8										
1091110	0550	15120		9.8	31.5		511		82.1			10.1	4.2	3.0	0.6
1091112	0552	15040		10.2	31.9		500		76.3			15.8	4.7	2.4	0.8
1091112	1314	18490	4.09	11.2	34.6	84.6	556		74.4			16.5	5.3	3.2	0.6
1091116	0558	15030		9.8			531		77.7			13.3	6.0	2.3	0.7
1091119	0612	18370		10.2			557		82.1			10.5	5.4	1.2	0.8
1091123	0614	18080		9.5			440		84.0			10.2	4.6	0.9	0.3
1091125	1730	25880		9.6			503		89.6			6.9	3.3	0.0	0.2

DATE	TIME	NA	K	CL	CA	BUN	CREAT	Mg	P	CRP	ESR	PCT	LIPASE	LACTATE
1091019	1713									0.231				
1091021	2009	138	3.1			13	0.68							
1091023	0923	140	3.4			8	0.87							
1091024	1918													26.0
1091024	1922	136	3.9			19	0.74			18.108				
1091025	0408	138	4.2	106	8.6	18	0.72							37.2
1091025	1246	139	3.9	107	7.4	20	0.80							
1091025	1247													36.4
1091025	1429		3.5											
1091025	2001													20.1
1091025	2003	141	3.6				0.83							
1091025	2311		3.6											14.6
1091026	0528													12.6
1091026	0531	139	3.2	104	7.3	24	0.98							
1091026	1443													8.2
1091026	1447		3.2											
1091026	1504							1.8	1.9					
1091026	2307													11.6
1091026	2308		3.1											
1091027	0621													16.7
1091027	0630	137	3.9	105	7.1	23	0.91							
1091027	1408													10.2
1091027	1416		3.6											
1091027	2125													13.5
1091027	2126		3.9											
1091028	0547	138	3.5	103	7.1		0.76	1.7	0.8					
1091028	0548													14.9
1091028	1415		3.7											
1091028	2253		4.1											
1091029	0552													16.4
1091029	0603	137	4.1											
1091029	1127							1.7	2.3					
1091030	0508													13.1
1091030	0509	138	4.5		7.3	22	0.81							
1091030	0650		3.9											
1091031	0645		4.3											
1091031	0646													11.1
1091101	0655	142	4.3	105	8.0	26	0.78	1.9	3.0					
1091102	0508													10.8
1091102	0509	141	3.7			28	0.72			12.772				
1091104	0616													22.7
1091104	0630	145	3.7	105	8.0	32	0.76							
1091105	0515													8.4
1091105	0516	146	3.6			31	0.68							
1091106	0513		3.8											
1091107	0552	142	3.1	104	8.6	33	0.81							
1091107	0553													16.6
1091107	2212		3.8		9.1			2.4	2.6					
1091108	0508	142	3.8											
1091108	1932	142	4.3		8.8			2.5	3.3					
1091110	0550	136	4.4	105	8.7	36	0.65							
1091110	0552													13.8
1091112	0607	134	5.0	101	9.4	33	0.60							
1091112	1315	140	4.9				0.53							

1091116	0602	134	4.9		8.9	27	0.52							
1091116	0603												132	
1091117	1226													20.0
1091117	1227	138	5.2		9.8			2.3						
1091119	0614											0.143		
1091119	0718	138	4.4		9.1	29	0.48							
1091123	0616													7.7
1091123	0630	137	3.2		8.9	29	0.53							
1091124	1000													13.8
1091124	1015		2.7		7.8			1.7						
1091125	1738											0.675		
1091125	1848													28.0
1091125	2028	141	4.9		8.8	69	0.76	2.6						
1091126	0805													14.0

DATE	TIME	GLUCOSE	PAB	TRF	ZN	CU	KETONE	CK	CKMB	PT-P	PT-C	APTT-P	APTT-C	TRO-T
1091025	0509	113												
1091025	1435									14.9	10.3	44.8		
1091027	1107		0.0590	1.2408										
1091027	2126													462.60
1091027	2127							33	3					
1091027	2358								3					482.50
1091106	0510									12.2	10.3	30.8		
1091112	1343		0.2124											
1091116	0555									11.5	10.3	30.8		
1091116	0910		0.1586											
1091117	1227							25						
1091117	1335								5					56.85
1091123	0927		0.1016											
1091124	1015							15						
1091124	1020								5					76.67
1091125	1737								6					
1091125	1741													51.94
1091125	2028							18						

DATE	TIME	SP-GR	PH	PROT	GLUC	KETON	BILI	UROB	OB	NITR	LEU	RBC	WBC	EPCEL
1091025	2112	1.020	5.0	1+	250.0	Trace	Negative	0.2	Trace	Negative	Negative	0-2	0-2	0-1
1091030	1038	1.013	5.5	1+	500.0	Negative	Negative	0.2	Negative	Negative	Negative	2-5	0-2	0-1
1091123	1953	1.018	5.0	3+	Negative	Negative	Negative	0.2	3+	Negative	2+	20-35	35-50	0-1

DATE	TIME	STOOL-OB	FAT	RBC	WBC	PARA
1091102	0939	2+				
1091107	1542	Trace				
1091110	1507			5 - 10	10 - 20	Not Found
1091123	1630			-	-	Not Found

DATE	TIME	PH	PCO2	PO2	SO2	BEB	HCO3	TCO2	HGB	FIO2
1091024	2107	7.474	33.0	73.8	95.7	0.8	23.7	24.7		
1091025	0408	7.362	41.2	54.0	86.0	-2.4	22.9	24.1		
1091025	1815	7.300	38.0	41.1	68.8	-7.5	18.2	19.4		
1091025	2000	7.243	55.8	48.8	75.5	-4.6	23.5	25.2		
1091028	0543	7.517	26.8	95.9	98.2	-0.1	21.2	22.1		
1091101	0650	7.443	35.9	93.0	97.5	0.4	24.0	25.1		
1091112	1312	7.426	35.4	131.4	99.0	-1.0	22.7	23.8		
1091124	1000	7.482	35.2	97.7	98.1	2.7	25.8	26.8		
1091125	1733	7.462	36.2	59.4	92.1	1.8	25.3	26.4		
1091126	1335	7.356	57.1	38.1	70.1	4.1	31.3	33.1		

DATE	TIME	PH	BEB	SCVO2	PO2	HCO3	HGB	PCO2	TCO2
1091117	1226	7.384	0.4	48.9	27.1	25.8		44.1	27.1
1091126	0758	7.353	4.8	46.0	26.5	32.2		59.2	34.0

特殊檢查

PFT :檢驗項目 **:FVC** 報告日期 **:1091014**

BMI : 17.17
Result:
PULMONARY LABORATORY
F-V LOOP PRE-RX POST-RX
SPIROMETRY (BTPS) PRED ACTUAL %PRED ACTUAL%PRED%CHG
FVC LITERS 2.13 0.88 41
FEV1 LITERS 1.72 0.80 47
FEV1/FVC % N/A 91
FEF25-75% L/SEC 1.70 0.84 50
FEF25% L/SEC 4.24 1.21 29
FEF50% L/SEC 2.22 1.01 45
FEF75% L/SEC 0.59 0.53 90
FEF200-1200 L/SEC
PEF L/SEC 4.52 1.24 27
FIVC LITTERS 2.13 0.73 34
FIF50% L/SEC N/A 0.66
PIF L/SEC N/A 0.73
FEF50/FIF50 UNITLESS N/A 1.53
MVV
INTERPRETATION:
CONCLUSION :
Poor effort, Poor cooperate. 《 病患無法配合FVC檢查 》

檢驗項目 **:PELVIS (PORTABLE)** 報告日期 **:1091108**
主 訴:
Result:
Finding:
\> Nonspecific bowel gas pattern
\> Partially obscured margin of bilateral psoas muscle and bilateral kidney shadows due to bowel gas
\> Increase in radiopacity of abdomen and thickness of paracolic gutters, ascites is considered.
\> Generalized osteoporosis
\> Degenerative change of thoracic and lumbar spine with marginal spur formation
\> Loss of height in the vertebral body of T12 is found, r/i compression fracture.
\> Surgical clips retention over abdomen and surgical autosuture retention over pelvic region
\> On Foley catheter, NG tube
\> Ostomy over RLQ of abdomen
Impression:
As above description.

檢驗項目 **:CHEST AP VIEW (PORTABLE)** 報告日期 **:1091103**
主 訴:
Result:
Finding:
\> Cardiomegaly with bilateral hilum prominence
\> Tortuosity of aorta with calcification
\> Haziness opacity in bilateral lower lung field with increased interstitial infiltration over residual lung field.
\> DJD of T-L spine with marginal spur formation
\> On endotracheal tube, nasogastric tube, central line, on EKG lead
\> R/O compression fracture of T12

Impression:
As above description.

檢驗項目 :**PELVIS (PORTABLE)** 報告日期 :**1091030**
主　訴 :
Result:
Finding:
> On N-J tube
> Retained surgical clips over abdomen
> Increased bowel gas with dilated bowel loops, R/O ileus or obstruction. Suggest clinical correlation and follow up.
> DJD change of T-L spine with spur formation
> R/I ascites
Impression:
As above description.

檢驗項目 :**PELVIS (PORTABLE)** 報告日期 :**1091103**
主　訴 :
Result:
Finding:
> Focal dilated small bowel loops, suggest clinical correlation and F/U.
> DJD change with osteophytes formation of visible T-L spine
> On nasogastric tube, Foley's catheter and EKG leads
> Surgical clips retention over mid abdomen and right abdomen
> S/P ostomy over RLQ abdomen
Impression:
As above description.

檢驗項目 :**ABDOMEN (KUB),SUPINE** 報告日期 :**1091103**
主　訴 :
Result:
Finding:
> Diffuse gaseous distension of bowel loop, ileus or obstruction should be considered. Suggest follow-up.
> Degenerative change of lumbar spine with osteophytes formation.
> Compression fracture of T12 vertebral body.
> Surgical clips retention over left middle and right lower abdomen.
> Surgical autosuture retention over pelvic region.
Impression:
As above description.

檢驗項目 :**CHEST AP VIEW (PORTABLE)** 報告日期 :**1091121**
主　訴 :
Result:
Finding:
> Borderline heart size
> Atherosclerotic change of the thoracic aorta
> Prominence of bilateral hila
> Multiple consolidative patches and hazy infiltrates in bilateral lungs
> Degenerative change of T- and L- spine with osteophyte formations
> Compression fracture of L1
> On endotracheal tube, nasogastric tube and EKG monitor
Impression:
As above description.

檢驗項目 :**CHEST AP VIEW (PORTABLE)** 報告日期 :**1091127**
主　訴 :
Result:
Finding:
> Borderline heart size
> Atherosclerotic change of the thoracic aorta
> Multiple consolidations and alveolar infiltrates in bilateral lungs
> Left C-P angle blunting may be due to pleural effusion or pleural change
> Degenerative change of T- and L- spine with osteophyte formations
> Suspicious compression fracture of T12
> On endotracheal tube, nasogastric tube and EKG monitor
Impression:
As above description

病理報告

- 活體組織切片病理報告　手術日期：**109/10/21**　病理號：**S0933404**

診斷醫師:陳律臻 報告日期:109/10/29
Pathologic diagnosis:
1. Rectum, laparoscopic low anterior resection -— Adenocarcinoma, moderately differentiated. Tumor invaded to muscularis propria. The bilateral cut ends and perirectal lymph nodes (0/21) are free of tumor.
2. IMA root, dissection -— Unremarkable (0/1).
3. Colon, cut end, distal, resection -— Unremarkable.
4. Small intestine, resection -— Congestion.

Ancillary study for diagnosis:
IHC stains for MLH1, MSH2, PMS2 and MSH6 are done on section L. All above markers are preserved.

Prognostic and predictive factor:
1. Tumor size: 3x2.8 cm.
2. Depth of invasion: Muscularis propria.
3. Histologic grade: G2: Moderately differentiated.
4. Angiolymphatic invasion: Not identified.
5. Perineural invasion: Not identified.
6. Tumor deposits (discontinuous extramural extension): Not identified.
7. Macroscopic tumor perforation: Not identified.
8. Surgical Margins: a) Proximal Margin: Uninvolved by invasive carcinoma, distance of tumor from margin: 10 cm. b) Distal Margin: Uninvolved by invasive carcinoma, distance of tumor from margin: 1.2 cm. c) Radial or Mesenteric Margin: Uninvolved by invasive carcinoma, distance of tumor from margin: more than 1 cm.
9. Lymph node status: Uninvolved (0/22).
10. Extranodal involvement: Not identified.
11. Tumor regression grading S/P CCRT: No known presurgical therapy.
12. Type of polyp in which invasive carcinoma arose: Not identified.
13. Additional pathologic findings: Not identified.
14. Pathological TNM Stage: pT2N0(According to the eighth edition, American Joint Committee on Cancer Staging Guidelines for Tumors).
15. TNM descriptors: Not applicable.

Gross description:
The specimen consists of 1) a segment of rectum, 15 cm in length and 3 cm in proximal cut end circumference, 2.5 cm in distal circumference, and perirectal soft tissue. A gray tan firm annular elevated and centrally ulcerative tumor, 3x2.8 cm, is noted, 1.2 cm to the nearest cut end. On cross section, the tumor invades to the

- muscular layer with more than 10 mm to the radial margin. The both cut ends are free of tumor grossly. 2) IMA root, a piece of tan yellow soft tissue, 6x4x1 cm. 3) distal cut end, a piece of tan mucosa-covered soft tissue, 2x1x0.5 cm. 4) a segment of small intesitne, 8 cm in length, 1.8 cm and 1 cm in bilateral circumference. No tumor found grossly. Representative part for section: A-B) specimen 2 C) specimen 3 all for section D-F) specimen 4: D) both cut ends E-F) small intestine G-P) specimen 5: G) proximal cut end H) tumor and distal cut end J) tumor and circumferential margin K) tumor L) tumor and normal M-P) lymph nodes.
  Note:
  1. The interpretation of MMR panel and recommended studies are as below: (1) No loss of nuclear expression of MMR proteins: low probability of microsatellite instability-high (MSI-H) . (2) Loss of nuclear expression of MLH1 and PMS2: testing for methylation of the MLH1 promoter and/or mutation of BRAF is indicated (the presence of a BRAF V600E mutation and/or MLH1 methylation suggests that the tumor is sporadic and germline evaluation is probably not indicated; absence of both MLH1 methylation and of BRAF V600E mutation suggests the possibility of Lynch syndrome, and sequencing and/or large deletion/duplication testing of germline MLH1 may be indicated). (3) Loss of nuclear expression of MSH2 and MSH6: high probability of Lynch syndrome (sequencing and/or large deletion/duplication testing of germline MSH2 may be indicated, and, if negative, sequencing and/or large deletion/duplication testing of germline MSH6 may be indicated). (4) Loss of nuclear expression of MSH6 only: high probability of Lynch syndrome (sequencing and/or large deletion/duplication testing of germline MSH6 may be indicated). (5) Loss of nuclear expression of PMS2 only: high probability of Lynch syndrome (sequencing and/or large deletion/duplication testing of germline PMS2 may be indicated). (6) Please note: There are exceptions to the above IHC interpretations. These results should not be considered in isolation, and clinical correlation with genetic counseling is recommended to assess the need for germline testing.
  2. This case has been peer reviewed by two doctors.
  #T-59300_2 #M-81403_2 2 1105 M0000F
  #T-C4560_2 #M-09183_2 0 1105 000000

其它

ORDINARY CULTURE I

DATE	RESULT-POSITIVE-1	RESULT-POSITIVE-2	SPECIMEN
1091025	Escherichia coli		Sputum
1091030	Escherichia coli		Sputum
1091110	Klebsiella pneumoniae		Sputum
1091112	Klebsiella pneumoniae		Sputum
1091123	Klebsiella pneumoniae		Sputum

ORDINARY CULTURE III

DATE	RESULT-NEGATIVE	SPECIMEN
1091124	No growth for ordinary culture.	Urine-indwelling catheter
1091104	Normal mixed flora presented.	Sputum
1091102	Normal mixed flora presented.	Sputum
1091028	No growth for ordinary culture.	Sputum
1091026	No growth for ordinary culture.	Urine-indwelling catheter
1091026	Normal mixed flora presented.	Sputum

ORDINARY CULTURE III

DATE	RESULT-NEGATIVE	SPECIMEN
1091110	Culture negative for Clostridium difficile	Stool

檢驗項目 :Screen for VRE in Fecal Culture 檢體:Anus 報告日期 :1091114
附註 :
陰性結果 : No vancomycin-resistant Enterococcus organisms isolated
陽性結果 : ISOLATED ORGANISM: COLONY COUNT:( )/ml
(1)
(2)
(3)
菌落量定義 : P:Pure H:Heavy M:Moderate S:Slight moderate F:Few E:Enrich
SUSCEPTIBILITY TEST: S:Susceptible I:Intermediate R:Resistant D: Susceptible Dose Dependent (SDD) NS: Nonsusceptible

	(1)	(2)	(3)	(4)
Ampicillin				
Cefazolin				
Clindamycin				
Clarithromycin				
Gentamicin				
Amikacin				
Chloramphenicol				
Ampicillin/Sulbactam				
Penicillin				
Gentamicin(120ug)				
Trimethoprim+Sulfamethoxazole				
Vancomycin				
Oxacillin				
Cefpirome				
Ciprofloxacin				
Minocycline				
Carbapen (IPM)				
Cefoperazone/Sulbactam				
Cefotaxime				
Ceftazidime				
Cefepime				
Ceftriaxone				
Flomoxef				
Levofloxacin				
Teicoplanin				
Piperacillin/Tazobactam				
Cefmetazole				
Aztreonam				
Tigecycline				
Amoxicillin/Clavulanic acid				
Cefoxitin				
Metronidazole				
Carbapen (MPM)				
Erythromycin				
Moxifloxacin				
Linezolid				
Ertapenem				
Colistin				
Daptomycin				

REMARK
注意事項：

檢驗項目 :Screen for CRE in Fecal Culture 檢體:Anus 報告日期 :1091113
附註 ：
陰性結果： Negative for CRE screening
陽性結果： ISOLATED ORGANISM: COLONY COUNT:( )/ml
(1)
(2)
(3)
菌落量定義： P:Pure H:Heavy M:Moderate S:Slight moderate F:Few E:Enrich
SUSCEPTIBILITY TEST: S:Susceptible I:Intermediate R:Resistant D: Susceptible Dose Dependent (SDD) NS: Nonsusceptible

	(1)	(2)	(3)	(4)
Ampicillin				
Cefazolin				
Clindamycin				
Clarithromycin				
Gentamicin				
Amikacin				
Chloramphenicol				
Ampicillin/Sulbactam				
Penicillin				
Gentamicin(120ug)				
Trimethoprim+Sulfamethoxazole				
Vancomycin				
Oxacillin				
Cefpirome				
Ciprofloxacin				
Minocycline				
Carbapen (IPM)				
Cefoperazone/Sulbactam				
Cefotaxime				
Ceftazidime				
Cefepime				
Ceftriaxone				
Flomoxef				
Levofloxacin				
Teicoplanin				
Piperacillin/Tazobactam				
Cefmetazole				
Aztreonam				
Tigecycline				
Amoxicillin/Clavulanic acid				
Cefoxitin				
Metronidazole				
Carbapen (MPM)				
Erythromycin				
Moxifloxacin				
Linezolid				
Ertapenem				
Colistin				
Daptomycin				

REMARK
注意事項：

檢驗項目 :Bacterial Culture(Ordinary) 檢體:Sputum 報告日期 :1091112
附註 ：
陰性結果：
陽性結果： ISOLATED ORGANISM: COLONY COUNT:( )/ml
(1) Klebsiella pneumoniae M
(2)
(3)
菌落量定義： P:Pure H:Heavy M:Moderate S:Slight moderate F:Few E:Enrich
SUSCEPTIBILITY TEST: S:Susceptible I:Intermediate R:Resistant D: Susceptible Dose Dependent (SDD) NS: Nonsusceptible

	(1)	(2)	(3)	(4)
Ampicillin				
Cefazolin	S=4			
Clindamycin				
Clarithromycin				
Gentamicin	S=1			
Amikacin	S=2			
Chloramphenicol				
Ampicillin/Sulbactam	S=8			
Penicillin				
Gentamicin(120ug)				
Trimethoprim+Sulfamethoxazole	R>=320			
Vancomycin				
Oxacillin				
Cefpirome				
Ciprofloxacin	S=0.25			
Minocycline				
Carbapen (IPM)	S=0.25			
Cefoperazone/Sulbactam	S=8			
Cefotaxime				
Ceftazidime	S=0.25			
Cefepime	S=0.12			
Ceftriaxone	S=0.25			
Flomoxef	S=2			
Levofloxacin				
Teicoplanin				
Piperacillin/Tazobactam	S=8			
Cefmetazole				
Aztreonam				
Tigecycline				
Amoxicillin/Clavulanic acid				
Cefoxitin	S=4			
Metronidazole				
Carbapen (MPM)				
Erythromycin				
Moxifloxacin				
Linezolid				
Ertapenem	S=0.12			
Colistin				
Daptomycin				

REMARK
注意事項： For Enterobacteriaceae :
1.對於腸內菌屬的敏感性試驗，cefepime若採用CLSI M100-24的判讀標準，當試驗結果為敏感性時，其建議治療劑量為1gm Q12H；若以最低抑制濃度（MIC）的方法，結果為敏感劑量依賴性（susceptible-dose dependent；SDD）時，當MIC為 4 ug/mL時則建議劑量為1gm/Q8H或2gm/Q12H，若是MIC為8 ug/mL時或採用紙錠擴散法結果為SDD，則建議治療劑量為2gm/Q8H。
2.Cefazolin predict results for the oral cephalosporins agents (cefaclor, cephalexin) when

used for therapy of uncomplicated UTIs due to E. coli, K. pneumoniae, and P. mirabilis.
3.Cefoperazone/Sulbactam目前並無國際性的判讀標準，現行引用文獻的判讀規範，僅供臨床參考。

檢驗項目 :**GRAM STAIN** 檢體:**Sputum** 報告日期 :**1091111**
1) PMN >25 /LPF
2) Squamous epithelial cell 25 /LPF
3) Few yeast-like microorganisms
4) Few Gram negative bacilli
5)
6)
7)
8)
備註:
@ FEW: 1 - 5, MODERATE: 6 - 30, HEAVY: > 30

檢驗項目 :**Clostridium difficile Culture** 檢體:**Stool** 報告日期 :**1091116**
附註 :
陰性結果： Culture negative for Clostridium difficile
陽性結果： ISOLATED ORGANISM: COLONY COUNT:( )/ml
(1)
(2)
(3)
菌落量定義： P:Pure H:Heavy M:Moderate S:Slight moderate F:Few E:Enrich
SUSCEPTIBILITY TEST: S:Susceptible I:Intermediate R:Resistant D: Susceptible Dose Dependent (SDD) NS: Nonsusceptible

	(1)	(2)	(3)	(4)
Ampicillin				
Cefazolin				
Clindamycin				
Clarithromycin				
Gentamicin				
Amikacin				
Chloramphenicol				
Ampicillin/Sulbactam				
Penicillin				
Gentamicin(120ug)				
Trimethoprim+Sulfamethoxazole				
Vancomycin				
Oxacillin				
Cefpirome				
Ciprofloxacin				
Minocycline				
Carbapen (IPM)				
Cefoperazone/Sulbactam				
Cefotaxime				
Ceftazidime				
Cefepime				
Ceftriaxone				
Flomoxef				
Levofloxacin				
Teicoplanin				
Piperacillin/Tazobactam				
Cefmetazole				
Aztreonam				
Tigecycline				
Amoxicillin/Clavulanic acid				
Cefoxitin				
Metronidazole				
Carbapen (MPM)				
Erythromycin				
Moxifloxacin				
Linezolid				
Ertapenem				
Colistin				
Daptomycin				

REMARK
注意事項：

檢驗項目 :**Stool Culture** 檢體:**Stool** 報告日期 :**1091112**
附註 :
陰性結果： Neg. for Salmonella, Shigella and Vibrio
陽性結果： ISOLATED ORGANISM: COLONY COUNT:( )/ml
(1)
(2)
(3)
菌落量定義： P:Pure H:Heavy M:Moderate S:Slight moderate F:Few E:Enrich
SUSCEPTIBILITY TEST: S:Susceptible I:Intermediate R:Resistant D: Susceptible Dose Dependent (SDD) NS: Nonsusceptible

	(1)	(2)	(3)	(4)
Ampicillin				
Cefazolin				
Clindamycin				
Clarithromycin				
Gentamicin				
Amikacin				
Chloramphenicol				
Ampicillin/Sulbactam				
Penicillin				
Gentamicin(120ug)				
Trimethoprim+Sulfamethoxazole				
Vancomycin				
Oxacillin				
Cefpirome				
Ciprofloxacin				
Minocycline				
Carbapen (IPM)				
Cefoperazone/Sulbactam				
Cefotaxime				
Ceftazidime				
Cefepime				
Ceftriaxone				
Flomoxef				
Levofloxacin				
Teicoplanin				
Piperacillin/Tazobactam				
Cefmetazole				
Aztreonam				
Tigecycline				
Amoxicillin/Clavulanic acid				
Cefoxitin				
Metronidazole				
Carbapen (MPM)				

Erythromycin
Moxifloxacin
Linezolid
Ertapenem
Colistin
Daptomycin
REMARK
注意事項：

檢驗項目 :Bacterial Culture(Ordinary) 檢體:Sputum 報告日期 :1091106
附註 ：
陰性結果： Normal mixed flora presented.
陽性結果： ISOLATED ORGANISM: COLONY COUNT:( )/ml
(1)
(2)
(3)
菌落量定義： P:Pure H:Heavy M:Moderate S:Slight moderate F:Few E:Enrich
SUSCEPTIBILITY TEST: S:Susceptible I:Intermediate R:Resistant D: Susceptible Dose Dependent (SDD) NS: Nonsusceptible
(1) (2) (3) (4)
Ampicillin
Cefazolin
Clindamycin
Clarithromycin
Gentamicin
Amikacin
Chloramphenicol
Ampicillin/Sulbactam
Penicillin
Gentamicin(120ug)
Trimethoprim+Sulfamethoxazole
Vancomycin
Oxacillin
Cefpirome
Ciprofloxacin
Minocycline
Carbapen (IPM)
Cefoperazone/Sulbactam
Cefotaxime
Ceftazidime
Cefepime
Ceftriaxone
Flomoxef
Levofloxacin
Teicoplanin
Piperacillin/Tazobactam
Cefmetazole
Aztreonam
Tigecycline
Amoxicillin/Clavulanic acid
Cefoxitin
Metronidazole
Carbapen (MPM)
Erythromycin
Moxifloxacin
Linezolid
Ertapenem
Colistin
Daptomycin
REMARK
注意事項：

檢驗項目 :GRAM STAIN 檢體:Sputum 報告日期 :1091104
1) PMN >25 /LPF
2) Squamous epithelial cell 25 /LPF
3) Few Gram negative bacilli
4)
5)
6)
7)
8)
備註:
@ FEW: 1 - 5, MODERATE: 6 - 30, HEAVY: > 30

檢驗項目 :Blood Culture 檢體:Blood 報告日期 :1091109
附註 ：
陰性結果： No bacterial growth for 7 days.
陽性結果： ISOLATED ORGANISM: COLONY COUNT:( )/ml
(1)
(2)
(3)
菌落量定義： P:Pure H:Heavy M:Moderate S:Slight moderate F:Few E:Enrich
SUSCEPTIBILITY TEST: S:Susceptible I:Intermediate R:Resistant D: Susceptible Dose Dependent (SDD) NS: Nonsusceptible
(1) (2) (3) (4)
Ampicillin
Cefazolin
Clindamycin
Clarithromycin
Gentamicin
Amikacin
Chloramphenicol
Ampicillin/Sulbactam
Penicillin
Gentamicin(120ug)
Trimethoprim+Sulfamethoxazole
Vancomycin
Oxacillin
Cefpirome
Ciprofloxacin
Minocycline
Carbapen (IPM)
Cefoperazone/Sulbactam
Cefotaxime
Ceftazidime
Cefepime
Ceftriaxone
Flomoxef
Levofloxacin
Teicoplanin
Piperacillin/Tazobactam
Cefmetazole
Aztreonam

Tigecycline
Amoxicillin/Clavulanic acid
Cefoxitin
Metronidazole
Carbapen (MPM)
Erythromycin
Moxifloxacin
Linezolid
Ertapenem
Colistin
Daptomycin
REMARK
注意事項：

檢驗項目 :Bacterial Culture(Ordinary) 檢體:Sputum 報告日期 :1091104
附註 ：
陰性結果： Normal mixed flora presented.
陽性結果： ISOLATED ORGANISM: COLONY COUNT:( )/ml
(1)
(2)
(3)
菌落量定義： P:Pure H:Heavy M:Moderate S:Slight moderate F:Few E:Enrich
SUSCEPTIBILITY TEST: S:Susceptible I:Intermediate R:Resistant D: Susceptible Dose Dependent (SDD) NS: Nonsusceptible
(1) (2) (3) (4)
Ampicillin
Cefazolin
Clindamycin
Clarithromycin
Gentamicin
Amikacin
Chloramphenicol
Ampicillin/Sulbactam
Penicillin
Gentamicin(120ug)
Trimethoprim+Sulfamethoxazole
Vancomycin
Oxacillin
Cefpirome
Ciprofloxacin
Minocycline
Carbapen (IPM)
Cefoperazone/Sulbactam
Cefotaxime
Ceftazidime
Cefepime
Ceftriaxone
Flomoxef
Levofloxacin
Teicoplanin
Piperacillin/Tazobactam
Cefmetazole
Aztreonam
Tigecycline
Amoxicillin/Clavulanic acid
Cefoxitin
Metronidazole
Carbapen (MPM)
Erythromycin
Moxifloxacin
Linezolid
Ertapenem
Colistin
Daptomycin
REMARK
注意事項：

檢驗項目 :GRAM STAIN 檢體:Sputum 報告日期 :1091103
1) PMN >25 /LPF
2) Squamous epithelial cell 25 /LPF
3) Few yeast-like microorganisms
4)
5)
6)
7)
8)
備註:
@ FEW: 1 - 5, MODERATE: 6 - 30, HEAVY: > 30

檢驗項目 :Blood Culture 檢體:Blood 報告日期 :1091105
附註 ：
陰性結果： No bacterial growth for 7 days.
陽性結果： ISOLATED ORGANISM: COLONY COUNT:( )/ml
(1)
(2)
(3)
菌落量定義： P:Pure H:Heavy M:Moderate S:Slight moderate F:Few E:Enrich
SUSCEPTIBILITY TEST: S:Susceptible I:Intermediate R:Resistant D: Susceptible Dose Dependent (SDD) NS: Nonsusceptible
(1) (2) (3) (4)
Ampicillin
Cefazolin
Clindamycin
Clarithromycin
Gentamicin
Amikacin
Chloramphenicol
Ampicillin/Sulbactam
Penicillin
Gentamicin(120ug)
Trimethoprim+Sulfamethoxazole
Vancomycin
Oxacillin
Cefpirome
Ciprofloxacin
Minocycline
Carbapen (IPM)
Cefoperazone/Sulbactam
Cefotaxime
Ceftazidime
Cefepime
Ceftriaxone
Flomoxef

Levofloxacin
Teicoplanin
Piperacillin/Tazobactam
Cefmetazole
Aztreonam
Tigecycline
Amoxicillin/Clavulanic acid
Cefoxitin
Metronidazole
Carbapen (MPM)
Erythromycin
Moxifloxacin
Linezolid
Ertapenem
Colistin
Daptomycin
REMARK
注意事項：

檢驗項目 :Bacterial Culture(Ordinary) 檢體:Sputum 報告日期 :1091102
附註 ：
陰性結果：
陽性結果： ISOLATED ORGANISM: COLONY COUNT:( )/ml
(1) Escherichia coli S
(2)
(3)
菌落量定義： P:Pure H:Heavy M:Moderate S:Slight moderate F:Few E:Enrich
SUSCEPTIBILITY TEST: S:Susceptible I:Intermediate R:Resistant D: Susceptible Dose Dependent (SDD) NS: Nonsusceptible

	(1)	(2)	(3)	(4)
Ampicillin				
Cefazolin	S=4			
Clindamycin				
Clarithromycin				
Gentamicin	S=1			
Amikacin	S=2			
Chloramphenicol				
Ampicillin/Sulbactam	S=2			
Penicillin				
Gentamicin(120ug)				
Trimethoprim+Sulfamethoxazole	S=20			
Vancomycin				
Oxacillin				
Cefpirome				
Ciprofloxacin	S=0.25			
Minocycline				
Carbapen (IPM)	S=0.25			
Cefoperazone/Sulbactam	S=8			
Cefotaxime				
Ceftazidime	S=0.12			
Cefepime	S=0.12			
Ceftriaxone	S=0.25			
Flomoxef	S=2			
Levofloxacin				
Teicoplanin				
Piperacillin/Tazobactam	S=4			
Cefmetazole				
Aztreonam				
Tigecycline				
Amoxicillin/Clavulanic acid				
Cefoxitin	S=4			
Metronidazole				
Carbapen (MPM)				
Erythromycin				
Moxifloxacin				
Linezolid				
Ertapenem	S=0.12			
Colistin				
Daptomycin				
REMARK				

注意事項： For Enterobacteriaceae :
1.對於腸內菌屬的敏感性試驗，cefepime若採用CLSI M100-24的判讀標準，當試驗結果為敏感性時，其建議治療劑量為1gm Q12H；若以最低抑制濃度（MIC）的方法，結果為敏感劑量依賴性（susceptible-dose dependent；SDD）時，當MIC為 4 ug/mL時則建議劑量為1gm/Q8H或2gm/Q12H，若是MIC為8 ug/mL時或採用紙錠擴散法結果為SDD，則建議治療劑量為2gm/Q8H。
2.Cefazolin predict results for the oral cephalosporins agents (cefaclor, cephalexin) when used for therapy of uncomplicated UTIs due to E. coli, K. pneumoniae, and P. mirabilis.
3.Cefoperazone/Sulbactam目前並無國際性的判讀標準，現行引用文獻的判讀規範，僅供臨床參考。

檢驗項目 :GRAM STAIN 檢體:Sputum 報告日期 :1091030
1) PMN >25 /LPF
2) Squamous epithelial cell 25 /LPF
3) No bacterium was found.
4)
5)
6)
7)
8)
備註:
@ FEW: 1 - 5, MODERATE: 6 - 30, HEAVY: > 30

檢驗項目 :Blood Culture 檢體:Blood 報告日期 :1091105
附註 ：
陰性結果： No bacterial growth for 7 days.
陽性結果： ISOLATED ORGANISM: COLONY COUNT:( )/ml
(1)
(2)
(3)
菌落量定義： P:Pure H:Heavy M:Moderate S:Slight moderate F:Few E:Enrich
SUSCEPTIBILITY TEST: S:Susceptible I:Intermediate R:Resistant D: Susceptible Dose Dependent (SDD) NS: Nonsusceptible

	(1)	(2)	(3)	(4)
Ampicillin				
Cefazolin				
Clindamycin				
Clarithromycin				
Gentamicin				
Amikacin				
Chloramphenicol				
Ampicillin/Sulbactam				
Penicillin				

Gentamicin(120ug)
Trimethoprim+Sulfamethoxazole
Vancomycin
Oxacillin
Cefpirome
Ciprofloxacin
Minocycline
Carbapen (IPM)
Cefoperazone/Sulbactam
Cefotaxime
Ceftazidime
Cefepime
Ceftriaxone
Flomoxef
Levofloxacin
Teicoplanin
Piperacillin/Tazobactam
Cefmetazole
Aztreonam
Tigecycline
Amoxicillin/Clavulanic acid
Cefoxitin
Metronidazole
Carbapen (MPM)
Erythromycin
Moxifloxacin
Linezolid
Ertapenem
Colistin
Daptomycin
REMARK
注意事項：

檢驗項目 :**Bacterial Culture(Ordinary)** 檢體:**Sputum** 報告日期 :**1091030**
附註 :
陰性結果： No growth for ordinary culture.
陽性結果： ISOLATED ORGANISM: COLONY COUNT:( )/ml
(1)
(2)
(3)
菌落量定義： P:Pure H:Heavy M:Moderate S:Slight moderate F:Few E:Enrich
SUSCEPTIBILITY TEST: S:Susceptible I:Intermediate R:Resistant D: Susceptible Dose Dependent (SDD) NS: Nonsusceptible
(1) (2) (3) (4)
Ampicillin
Cefazolin
Clindamycin
Clarithromycin
Gentamicin
Amikacin
Chloramphenicol
Ampicillin/Sulbactam
Penicillin
Gentamicin(120ug)
Trimethoprim+Sulfamethoxazole
Vancomycin
Oxacillin
Cefpirome
Ciprofloxacin
Minocycline
Carbapen (IPM)
Cefoperazone/Sulbactam
Cefotaxime
Ceftazidime
Cefepime
Ceftriaxone
Flomoxef
Levofloxacin
Teicoplanin
Piperacillin/Tazobactam
Cefmetazole
Aztreonam
Tigecycline
Amoxicillin/Clavulanic acid
Cefoxitin
Metronidazole
Carbapen (MPM)
Erythromycin
Moxifloxacin
Linezolid
Ertapenem
Colistin
Daptomycin
REMARK
注意事項：

檢驗項目 :**GRAM STAIN** 檢體:**Sputum** 報告日期 :**1091028**
1) PMN >25 /LPF
2) Squamous epithelial cell 25 /LPF
3) No bacterium was found.
4)
5)
6)
7)
8)
備註:
@ FEW: 1 - 5, MODERATE: 6 - 30, HEAVY: > 30

檢驗項目 :**Bacterial Culture(Ordinary)** 檢體:**Sputum** 報告日期 :**1091028**
附註 :
陰性結果： Normal mixed flora presented.
陽性結果： ISOLATED ORGANISM: COLONY COUNT:( )/ml
(1)
(2)
(3)
菌落量定義： P:Pure H:Heavy M:Moderate S:Slight moderate F:Few E:Enrich
SUSCEPTIBILITY TEST: S:Susceptible I:Intermediate R:Resistant D: Susceptible Dose Dependent (SDD) NS: Nonsusceptible
(1) (2) (3) (4)
Ampicillin
Cefazolin
Clindamycin
Clarithromycin

Gentamicin
Amikacin
Chloramphenicol
Ampicillin/Sulbactam
Penicillin
Gentamicin(120ug)
Trimethoprim+Sulfamethoxazole
Vancomycin
Oxacillin
Cefpirome
Ciprofloxacin
Minocycline
Carbapen (IPM)
Cefoperazone/Sulbactam
Cefotaxime
Ceftazidime
Cefepime
Ceftriaxone
Flomoxef
Levofloxacin
Teicoplanin
Piperacillin/Tazobactam
Cefmetazole
Aztreonam
Tigecycline
Amoxicillin/Clavulanic acid
Cefoxitin
Metronidazole
Carbapen (MPM)
Erythromycin
Moxifloxacin
Linezolid
Ertapenem
Colistin
Daptomycin
REMARK
注意事項：

檢驗項目 :**GRAM STAIN** 檢體:**Sputum** 報告日期 :**1091026**
1) PMN >25 /LPF
2) Squamous epithelial cell 25 /LPF
3) Few Gram negative bacilli
4)
5)
6)
7)
8)
備註:
@ FEW: 1 - 5, MODERATE: 6 - 30, HEAVY: > 30

檢驗項目 :**Bacterial Culture(Ordinary)** 檢體:**Sputum** 報告日期 :**1091028**
附註 ：
陰性結果： Normal mixed flora presented.
陽性結果： ISOLATED ORGANISM: COLONY COUNT:( )/ml
(1)
(2)
(3)
菌落量定義： P:Pure H:Heavy M:Moderate S:Slight moderate F:Few E:Enrich
SUSCEPTIBILITY TEST: S:Susceptible I:Intermediate R:Resistant D: Susceptible Dose Dependent (SDD) NS: Nonsusceptible
(1) (2) (3) (4)
Ampicillin
Cefazolin
Clindamycin
Clarithromycin
Gentamicin
Amikacin
Chloramphenicol
Ampicillin/Sulbactam
Penicillin
Gentamicin(120ug)
Trimethoprim+Sulfamethoxazole
Vancomycin
Oxacillin
Cefpirome
Ciprofloxacin
Minocycline
Carbapen (IPM)
Cefoperazone/Sulbactam
Cefotaxime
Ceftazidime
Cefepime
Ceftriaxone
Flomoxef
Levofloxacin
Teicoplanin
Piperacillin/Tazobactam
Cefmetazole
Aztreonam
Tigecycline
Amoxicillin/Clavulanic acid
Cefoxitin
Metronidazole
Carbapen (MPM)
Erythromycin
Moxifloxacin
Linezolid
Ertapenem
Colistin
Daptomycin
REMARK
注意事項：

檢驗項目 :**GRAM STAIN** 檢體:**Sputum** 報告日期 :**1091026**
1) PMN 25 /LPF
2) Squamous epithelial cell 25 /LPF
3) No bacterium was found.
4)
5)
6)
7)
8)
備註:

@ FEW: 1 - 5, MODERATE: 6 - 30, HEAVY: > 30

檢驗項目 :Bacterial Culture(Ordinary) 檢體:Urine-indwelling catheter 報告日期 :1091028
附註 :
陰性結果： No growth for ordinary culture.
陽性結果： ISOLATED ORGANISM: COLONY COUNT:( )/ml
(1)
(2)
(3)
菌落量定義： P:Pure H:Heavy M:Moderate S:Slight moderate F:Few E:Enrich
SUSCEPTIBILITY TEST: S:Susceptible I:Intermediate R:Resistant D: Susceptible Dose Dependent (SDD) NS: Nonsusceptible
(1) (2) (3) (4)
Ampicillin
Cefazolin
Clindamycin
Clarithromycin
Gentamicin
Amikacin
Chloramphenicol
Ampicillin/Sulbactam
Penicillin
Gentamicin(120ug)
Trimethoprim+Sulfamethoxazole
Vancomycin
Oxacillin
Cefpirome
Ciprofloxacin
Minocycline
Carbapen (IPM)
Cefoperazone/Sulbactam
Cefotaxime
Ceftazidime
Cefepime
Ceftriaxone
Flomoxef
Levofloxacin
Teicoplanin
Piperacillin/Tazobactam
Cefmetazole
Aztreonam
Tigecycline
Amoxicillin/Clavulanic acid
Cefoxitin
Metronidazole
Carbapen (MPM)
Erythromycin
Moxifloxacin
Linezolid
Ertapenem
Colistin
Daptomycin
REMARK
注意事項：

檢驗項目 :Screen for VRE in Fecal Culture 檢體:Anus 報告日期 :1091028
附註 :
陰性結果： No vancomycin-resistant Enterococcus organisms isolated
陽性結果： ISOLATED ORGANISM: COLONY COUNT:( )/ml
(1)
(2)
(3)
菌落量定義： P:Pure H:Heavy M:Moderate S:Slight moderate F:Few E:Enrich
SUSCEPTIBILITY TEST: S:Susceptible I:Intermediate R:Resistant D: Susceptible Dose Dependent (SDD) NS: Nonsusceptible
(1) (2) (3) (4)
Ampicillin
Cefazolin
Clindamycin
Clarithromycin
Gentamicin
Amikacin
Chloramphenicol
Ampicillin/Sulbactam
Penicillin
Gentamicin(120ug)
Trimethoprim+Sulfamethoxazole
Vancomycin
Oxacillin
Cefpirome
Ciprofloxacin
Minocycline
Carbapen (IPM)
Cefoperazone/Sulbactam
Cefotaxime
Ceftazidime
Cefepime
Ceftriaxone
Flomoxef
Levofloxacin
Teicoplanin
Piperacillin/Tazobactam
Cefmetazole
Aztreonam
Tigecycline
Amoxicillin/Clavulanic acid
Cefoxitin
Metronidazole
Carbapen (MPM)
Erythromycin
Moxifloxacin
Linezolid
Ertapenem
Colistin
Daptomycin
REMARK
注意事項：

檢驗項目 :Screen for CRE in Fecal Culture 檢體:Anus 報告日期 :1091027
附註 :
陰性結果： Negative for CRE screening
陽性結果： ISOLATED ORGANISM: COLONY COUNT:( )/ml
(1)

(2)
(3)
菌落量定義： P:Pure H:Heavy M:Moderate S:Slight moderate F:Few E:Enrich
SUSCEPTIBILITY TEST: S:Susceptible I:Intermediate R:Resistant D: Susceptible Dose Dependent (SDD) NS: Nonsusceptible

	(1)	(2)	(3)	(4)
Ampicillin				
Cefazolin				
Clindamycin				
Clarithromycin				
Gentamicin				
Amikacin				
Chloramphenicol				
Ampicillin/Sulbactam				
Penicillin				
Gentamicin(120ug)				
Trimethoprim+Sulfamethoxazole				
Vancomycin				
Oxacillin				
Cefpirome				
Ciprofloxacin				
Minocycline				
Carbapen (IPM)				
Cefoperazone/Sulbactam				
Cefotaxime				
Ceftazidime				
Cefepime				
Ceftriaxone				
Flomoxef				
Levofloxacin				
Teicoplanin				
Piperacillin/Tazobactam				
Cefmetazole				
Aztreonam				
Tigecycline				
Amoxicillin/Clavulanic acid				
Cefoxitin				
Metronidazole				
Carbapen (MPM)				
Erythromycin				
Moxifloxacin				
Linezolid				
Ertapenem				
Colistin				
Daptomycin				
REMARK				

注意事項：

檢驗項目 :Blood Culture 檢體:Blood 報告日期 :1091101
附註 ：
陰性結果： No bacterial growth for 7 days.
陽性結果： ISOLATED ORGANISM: COLONY COUNT:( )/ml
(1)
(2)
(3)
菌落量定義： P:Pure H:Heavy M:Moderate S:Slight moderate F:Few E:Enrich
SUSCEPTIBILITY TEST: S:Susceptible I:Intermediate R:Resistant D: Susceptible Dose Dependent (SDD) NS: Nonsusceptible

	(1)	(2)	(3)	(4)
Ampicillin				
Cefazolin				
Clindamycin				
Clarithromycin				
Gentamicin				
Amikacin				
Chloramphenicol				
Ampicillin/Sulbactam				
Penicillin				
Gentamicin(120ug)				
Trimethoprim+Sulfamethoxazole				
Vancomycin				
Oxacillin				
Cefpirome				
Ciprofloxacin				
Minocycline				
Carbapen (IPM)				
Cefoperazone/Sulbactam				
Cefotaxime				
Ceftazidime				
Cefepime				
Ceftriaxone				
Flomoxef				
Levofloxacin				
Teicoplanin				
Piperacillin/Tazobactam				
Cefmetazole				
Aztreonam				
Tigecycline				
Amoxicillin/Clavulanic acid				
Cefoxitin				
Metronidazole				
Carbapen (MPM)				
Erythromycin				
Moxifloxacin				
Linezolid				
Ertapenem				
Colistin				
Daptomycin				
REMARK				

注意事項：

檢驗項目 :Blood Culture 檢體:Blood-from catheter 報告日期 :1091101
附註 ：
陰性結果： No bacterial growth for 7 days.
陽性結果： ISOLATED ORGANISM: COLONY COUNT:( )/ml
(1)
(2)
(3)
菌落量定義： P:Pure H:Heavy M:Moderate S:Slight moderate F:Few E:Enrich
SUSCEPTIBILITY TEST: S:Susceptible I:Intermediate R:Resistant D: Susceptible Dose Dependent (SDD) NS: Nonsusceptible

	(1)	(2)	(3)	(4)
Ampicillin				

Cefazolin
Clindamycin
Clarithromycin
Gentamicin
Amikacin
Chloramphenicol
Ampicillin/Sulbactam
Penicillin
Gentamicin(120ug)
Trimethoprim+Sulfamethoxazole
Vancomycin
Oxacillin
Cefpirome
Ciprofloxacin
Minocycline
Carbapen (IPM)
Cefoperazone/Sulbactam
Cefotaxime
Ceftazidime
Cefepime
Ceftriaxone
Flomoxef
Levofloxacin
Teicoplanin
Piperacillin/Tazobactam
Cefmetazole
Aztreonam
Tigecycline
Amoxicillin/Clavulanic acid
Cefoxitin
Metronidazole
Carbapen (MPM)
Erythromycin
Moxifloxacin
Linezolid
Ertapenem
Colistin
Daptomycin
REMARK
注意事項：

檢驗項目 :Blood Culture 檢體:Blood 報告日期 :1091031
附註 ：
陰性結果： No bacterial growth for 7 days.
陽性結果： ISOLATED ORGANISM: COLONY COUNT:( )/ml
(1)
(2)
(3)
菌落量定義： P:Pure H:Heavy M:Moderate S:Slight moderate F:Few E:Enrich
SUSCEPTIBILITY TEST: S:Susceptible I:Intermediate R:Resistant D: Susceptible Dose Dependent (SDD) NS: Nonsusceptible
(1) (2) (3) (4)
Ampicillin
Cefazolin
Clindamycin
Clarithromycin
Gentamicin
Amikacin
Chloramphenicol
Ampicillin/Sulbactam
Penicillin
Gentamicin(120ug)
Trimethoprim+Sulfamethoxazole
Vancomycin
Oxacillin
Cefpirome
Ciprofloxacin
Minocycline
Carbapen (IPM)
Cefoperazone/Sulbactam
Cefotaxime
Ceftazidime
Cefepime
Ceftriaxone
Flomoxef
Levofloxacin
Teicoplanin
Piperacillin/Tazobactam
Cefmetazole
Aztreonam
Tigecycline
Amoxicillin/Clavulanic acid
Cefoxitin
Metronidazole
Carbapen (MPM)
Erythromycin
Moxifloxacin
Linezolid
Ertapenem
Colistin
Daptomycin
REMARK
注意事項：

檢驗項目 :Blood Culture 檢體:Blood 報告日期 :1091031
附註 ：
陰性結果： No bacterial growth for 7 days.
陽性結果： ISOLATED ORGANISM: COLONY COUNT:( )/ml
(1)
(2)
(3)
菌落量定義： P:Pure H:Heavy M:Moderate S:Slight moderate F:Few E:Enrich
SUSCEPTIBILITY TEST: S:Susceptible I:Intermediate R:Resistant D: Susceptible Dose Dependent (SDD) NS: Nonsusceptible
(1) (2) (3) (4)
Ampicillin
Cefazolin
Clindamycin
Clarithromycin
Gentamicin
Amikacin
Chloramphenicol
Ampicillin/Sulbactam

Penicillin
Gentamicin(120ug)
Trimethoprim+Sulfamethoxazole
Vancomycin
Oxacillin
Cefpirome
Ciprofloxacin
Minocycline
Carbapen (IPM)
Cefoperazone/Sulbactam
Cefotaxime
Ceftazidime
Cefepime
Ceftriaxone
Flomoxef
Levofloxacin
Teicoplanin
Piperacillin/Tazobactam
Cefmetazole
Aztreonam
Tigecycline
Amoxicillin/Clavulanic acid
Cefoxitin
Metronidazole
Carbapen (MPM)
Erythromycin
Moxifloxacin
Linezolid
Ertapenem
Colistin
Daptomycin
REMARK
注意事項：

檢驗項目 **:Blood Culture** 檢體**:Blood** 報告日期 **:1091031**
附註 ：
陰性結果： No bacterial growth for 7 days.
陽性結果： ISOLATED ORGANISM: COLONY COUNT:( )/ml
(1)
(2)
(3)
菌落量定義： P:Pure H:Heavy M:Moderate S:Slight moderate F:Few E:Enrich
SUSCEPTIBILITY TEST: S:Susceptible I:Intermediate R:Resistant D: Susceptible Dose Dependent (SDD) NS: Nonsusceptible

	(1)	(2)	(3)	(4)
Ampicillin				
Cefazolin				
Clindamycin				
Clarithromycin				
Gentamicin				
Amikacin				
Chloramphenicol				
Ampicillin/Sulbactam				
Penicillin				
Gentamicin(120ug)				
Trimethoprim+Sulfamethoxazole				
Vancomycin				
Oxacillin				
Cefpirome				
Ciprofloxacin				
Minocycline				
Carbapen (IPM)				
Cefoperazone/Sulbactam				
Cefotaxime				
Ceftazidime				
Cefepime				
Ceftriaxone				
Flomoxef				
Levofloxacin				
Teicoplanin				
Piperacillin/Tazobactam				
Cefmetazole				
Aztreonam				
Tigecycline				
Amoxicillin/Clavulanic acid				
Cefoxitin				
Metronidazole				
Carbapen (MPM)				
Erythromycin				
Moxifloxacin				
Linezolid				
Ertapenem				
Colistin				
Daptomycin				

REMARK
注意事項：

檢驗項目 **:Bacterial Culture(Ordinary)** 檢體**:Sputum** 報告日期 **:1091027**
附註 ：
陰性結果：
陽性結果： ISOLATED ORGANISM: COLONY COUNT:( )/ml
(1) Escherichia coli M
(2)
(3)
菌落量定義： P:Pure H:Heavy M:Moderate S:Slight moderate F:Few E:Enrich
SUSCEPTIBILITY TEST: S:Susceptible I:Intermediate R:Resistant D: Susceptible Dose Dependent (SDD) NS: Nonsusceptible

	(1)	(2)	(3)	(4)
Ampicillin				
Cefazolin	S=4			
Clindamycin				
Clarithromycin				
Gentamicin	S=1			
Amikacin	S=2			
Chloramphenicol				
Ampicillin/Sulbactam	S=2			
Penicillin				
Gentamicin(120ug)				
Trimethoprim+Sulfamethoxazole	S=20			
Vancomycin				
Oxacillin				
Cefpirome				
Ciprofloxacin	S=0.25			

Minocycline
Carbapen (IPM) S=0.25
Cefoperazone/Sulbactam S=8
Cefotaxime
Ceftazidime S=0.12
Cefepime S=0.12
Ceftriaxone S=0.25
Flomoxef S=2
Levofloxacin
Teicoplanin
Piperacillin/Tazobactam S=4
Cefmetazole
Aztreonam
Tigecycline
Amoxicillin/Clavulanic acid
Cefoxitin S=4
Metronidazole
Carbapen (MPM)
Erythromycin
Moxifloxacin
Linezolid
Ertapenem S=0.12
Colistin
Daptomycin
REMARK
注意事項： For Enterobacteriaceae :

1.對於腸內菌屬的敏感性試驗，cefepime若採用CLSI M100-24的判讀標準，當試驗結果為敏感性時，其建議治療劑量為1gm Q12H；若以最低抑制濃度（MIC）的方法，結果為敏感劑量依賴性（susceptible-dose dependent；SDD）時，當MIC為 4 ug/mL時則建議劑量為1gm/Q8H或2gm/Q12H，若是MIC為8 ug/mL時或採用紙錠擴散法結果為SDD，則建議治療劑量為2gm/Q8H。

2.Cefazolin predict results for the oral cephalosporins agents (cefaclor, cephalexin) when used for therapy of uncomplicated UTIs due to E. coli, K. pneumoniae, and P. mirabilis.

3.Cefoperazone/Sulbactam目前並無國際性的判讀標準，現行引用文獻的判讀規範，僅供臨床參考。

檢驗項目 :**Gram Stain** 檢體:**Sputum** 報告日期 :**1091026**
1) PMN >25 /LPF
2) Squamous epithelial cell 25 /LPF
3) Moderate Gram negative bacilli
4)
5)
6)
7)
8)
備註:
@ FEW: 1 - 5, MODERATE: 6 - 30, HEAVY: > 30

檢驗項目 :**Blood Culture** 檢體:**Blood** 報告日期 :**1060310**
附註 :
陰性結果： No bacterial growth for 7 days.
陽性結果： ISOLATED ORGANISM: COLONY COUNT:( )/ml
(1)
(2)
(3)
菌落量定義： P:Pure H:Heavy M:Moderate S:Slight moderate F:Few E:Enrich
SUSCEPTIBILITY TEST: S:Susceptible I:Intermediate R:Resistant D: Susceptible Dose Dependent (SDD) NS: Nonsusceptible
(1) (2) (3) (4)
Ampicillin
Cefazolin
Clindamycin
Clarithromycin
Gentamicin
Amikacin
Chloramphenicol
Ampicillin/Sulbactam
Penicillin
Gentamicin(120ug)
Trimethoprim+Sulfamethoxazole
Vancomycin
Oxacillin
Cefpirome
Ciprofloxacin
Minocycline
Carbapen (IPM)
Cefoperazone/Sulbactam
Cefotaxime
Ceftazidime
Cefepime
Ceftriaxone
Flomoxef
Levofloxacin
Teicoplanin
Piperacillin/Tazobactam
Cefmetazole
Aztreonam
Tigecycline
Amoxicillin/Clavulanic acid
Cefoxitin
Metronidazole
Carbapen (MPM)
Erythromycin
Moxifloxacin
Linezolid
Ertapenem
Colistin
Daptomycin
REMARK
注意事項：

檢驗項目 :**Blood Culture** 檢體:**Blood** 報告日期 :**1060310**
附註 :
陰性結果： No bacterial growth for 7 days.
陽性結果： ISOLATED ORGANISM: COLONY COUNT:( )/ml
(1)
(2)
(3)
菌落量定義： P:Pure H:Heavy M:Moderate S:Slight moderate F:Few E:Enrich
SUSCEPTIBILITY TEST: S:Susceptible I:Intermediate R:Resistant D: Susceptible Dose Dependent (SDD) NS: Nonsusceptible
(1) (2) (3) (4)
Ampicillin

Cefazolin
Clindamycin
Clarithromycin
Gentamicin
Amikacin
Chloramphenicol
Ampicillin/Sulbactam
Penicillin
Gentamicin(120ug)
Trimethoprim+Sulfamethoxazole
Vancomycin
Oxacillin
Cefpirome
Ciprofloxacin
Minocycline
Carbapen (IPM)
Cefoperazone/Sulbactam
Cefotaxime
Ceftazidime
Cefepime
Ceftriaxone
Flomoxef
Levofloxacin
Teicoplanin
Piperacillin/Tazobactam
Cefmetazole
Aztreonam
Tigecycline
Amoxicillin/Clavulanic acid
Cefoxitin
Metronidazole
Carbapen (MPM)
Erythromycin
Moxifloxacin
Linezolid
Ertapenem
Colistin
Daptomycin
REMARK
注意事項：

檢驗項目 :**Acid Fast Stain - Concentrated** 檢體:**Sputum** 報告日期 :**1091109**
Result: NOT FOUND
備註:

檢驗項目 :**Acid Fast Stain - Concentrated** 檢體:**Sputum** 報告日期 :**1091109**
Result: NOT FOUND
備註:

檢驗項目 :**Acid Fast Stain - Concentrated** 檢體:**Sputum** 報告日期 :**1091109**
Result: NOT FOUND
備註:

檢驗項目 :**Fungal Culture** 檢體:**Sputum** 報告日期 :**1091112**
陰性結果：
陽性結果 Candida albicans 菌落數||

	Amphotericin B	Anidulafugine	Caspofungine	Flucytosine
(1)				
(2)				
(3)				

	Fluconazole	Itraconazole	Ketoconazole	Micafungine
(1)				
(2)				
(3)				

	Posaconazole	Ravuconazole	Voriconazole
(1)			
(2)			
(3)			

SUSCEPTIBILITY TEST: S:Susceptible I:Intermediate R:Resistant D: Susceptible Dose Dependent (SDD) NS: Nonsusceptible
備註:

檢驗項目 :**Direct Microscopic Exam.** 檢體:**Sputum** 報告日期 :**1091102**
Result: KOH (+)
備註:

檢驗項目 :**Duodenoscopy (D-SCOPY)+PHOTO**胃鏡 報告日期 :**1091030**
Result:
ENDOSCOPY FINDINGS:
Esophagus: normal appearance
EC junction: mucosa break 5 mm
Fundus: hyperemia
Body: hyperemia
Angularis: hyperemia
Antrum: hyperemia
Pylorus: hyperemia
Duodenum: normal appearance
DIAGNOSIS/IMPRESSION:
1. Reflux esophagitis, LA, Gr A
ADDITIONAL PROCEDURE: NJ insertion from incisor site 90cm
COMPLICATIONS: There were no complications associated with the procedure.
RECOMMENDATION:
1. Follow KUB to check NJ position
CLINICAL DIAGNOSIS/SYMPTOMS:for NJ insertion
ENDOSCOPE CODE NO: G33
PREMEDICATION: Xylocain spray;gascon

檢驗項目 :**Latex test for Cryptococcus Antigen** 檢體:**Blood** 報告日期 :**1091119**
Result: Latex test for Cryptococcal： NEGATIVE
附註：

檢驗項目 :**COLOR DOPPLER + 2D** 報告日期 :**1091119**
檢查目的及臨床診斷：
Result:
TAPE NO. EP 7C > BEGINS > ENDS > WT: > KG
TAPE NO. > BEGINS > ENDS > BP: >/ > mmHG

COMMENTS： LA (4.3 CM) CHAMBER DILATATION
AORTIC VALVE THICKENED WITH MODERATE AR
THICKENED MV AND MITRAL ANNULAR CALCIFICATION INVOLVE THE MV
(TRAND MV PEAK/MEAN PG – 27/9 mmHG)
(MVA BY PHT: 2.4-2.75 CM**2) WITH MODERATE MR
MILD TO MODERATE TR WITH PEAK/MEAN SYSTOLIC PRESSURE
GRADIENT – 38/28 MMHG
SUGGEST PULMONARY HYPERTENSION
DILATED PA TRUNK (– 2.9 CM) WITH MILD PR
CONC. LVH (1.1, 1.2 CM)
NORMAL LV SYSTOLIC WALL MOTION.
THE LV EJECTION FRACTION IS 61 %
REVERSE MV E/A RATIO
MV E/E' SEPTAL = 36.4
MV E/E' LATERAL = 23.8
MINIMAL AMOUNT OF PERICARDIAL EFFUSION
END

出院時情況

- 21.下轉他院

出院計劃

- 服藥內容:

藥名	劑量	劑量單位	用法	頻次	數量	數量單位	現況	天數	單位
Cortisone tab 25mg	0.5	TAB	PO	QPM	1	TAB	TH	1	天
30 Novomix Flexpen	36	U	SC	30BBF	1	U	TH	1	天
Lactam tab 500mg	1	TAB	PO	Q6HPRN	4	TAB	TH	1	天
Cortisone tab 25mg	1	TAB	PO	QD	1	TAB	TH	1	天
Midodrine tab2.5mg	4	TAB	PO	QDPRN	4	TAB	TH	1	天
Acetylcysteine tab	1	TAB	PO	QD	1	TAB	TH	1	天
(ㄩ)Takepron OD tab 30mg	1	TAB	PO	QDAC	1	TAB	TH	1	天
Plavix tab 75mg	1	TAB	PO	QD	1	TAB	TH	1	天
Morphine inj 10mg	3	MG	SC	Q4HPRN	2	MG	TH	1	天
30 Novomix Flexpen	18	U	SC	30BD	1	U	TH	1	天

- 一般生活注意事項及衛教:
  - 遵從醫囑: 長期臥床病人，需幫助病人拍背以利咳痰。 早晚各量一次體溫，如有發燒狀況，請主動告知。 無法以嘴巴進食病人，需量使用鼻胃管灌食，以避免發生吸入性肺炎。 注意出血徵兆如：臉色蒼白、呼吸急促、心跳加快、煩燥不安、解黑便及嘔血。
- 緊急連絡電話:04-23592525 轉分機3872

主治醫師 1153B 王俊隆 / 住院醫師 1723B 王彥景

正式摘要(電子簽章：000000204203544)

RCC / RCC

doctorRank:B
